# Ratiometric Fluorescence Assay for Nitroreductase Activity: Locked-Flavylium Fluorophore as a NTR-Sensitive Molecular Probe

**DOI:** 10.3390/molecules26041088

**Published:** 2021-02-19

**Authors:** Su Jung Kim, Jung Won Yoon, Shin A Yoon, Min Hee Lee

**Affiliations:** Department of Chemistry, Sookmyung Women’s University, Seoul 04310, Korea; sujyungcc@sookmyung.ac.kr (S.J.K.); iyjw1118@sookmyung.ac.kr (J.W.Y.); dbstlsdk23@sookmyung.ac.kr (S.A.Y.)

**Keywords:** nitroreductase, ratiometric fluorescent molecule, bioimaging, cancer cells

## Abstract

Nitroreductases belong to a member of flavin-containing enzymes that can reduce nitroaromatic compounds to amino derivatives with NADH as an electron donor. NTR activity is known to be elevated in the cancerous environment and is considered an advantageous target in therapeutic prodrugs for the treatment of cancer. Here, we developed a ratiometric fluorescent molecule for observing NTR activity in living cells. This can provide a selective and sensitive response to NTR with a distinct increase in fluorescence ratio (FI_530_/FI_630_) as well as color changes. We also found a significant increase in NTR activity in cervical cancer HeLa and lung cancer A549 cells compared to non-cancerous NIH3T3. We proposed that this new ratiometric fluorescent molecule could potentially be used as a NTR-sensitive molecular probe in the field of cancer diagnosis and treatment development related to NTR activity.

## 1. Introduction

Nitroreductases (NTR) are flavoenzymes that catalyze the reduction of nitroaromatic compounds to amines using NADH as an electron donor [1,2]. NTR activity is important as a therapeutic target for biological detoxification of nitroaromatic compounds and related diseases [3]. NTR activity is especially significantly increased in hypoxic tumors and various cancers such as cervical, breast, liver, and lung cancers [4], indicating that NTR is a potential theranostic target for cancer [5,6]. Indeed, a method capable of the accurate and spatiotemporal detection of NTR-mediated enzyme reaction in the human live cells is an important key technology for the development of theranostics.

So far, we have used several analytical tools for the detection of NTR, such as nuclear magnetic resonance (NMR), electron paramagnetic resonance (EPR), Clark electrode [7,8,9]. However, these are limited to a spatiotemporal detection of enzyme reactions in the human live cells.

More recently, some fluorescent molecules have been developed to detect NTR enzymes in biological specimens and the live cells [10,11,12,13,14,15]. These provide rapid detection and biocompatibility of NTR activity in biological applications [16]. For example, Wang et al. developed a coumarin-based fluorescent probe capable of detection of NTR via a cascade cyclization reaction, which could be used for selectively detection of NTR in the biological system [11]. Moreover, Tang et al. reported a dual enzyme reaction-based probe consisting of *p*-nitrobenzene and trimethyl-locked quinone sensitive to NTR and human NAD(P)H quinone oxidoreductase-1 (hNQO1), respectively. It was able to precisely detect two enzyme activities and differentiate between the cancerous and normal cells [15]. However, the probes provided a fluorescent Off-On signal at relatively short excitation and emission wavelengths. Thus, it has inevitably been interfered with the assay-independent factors such as autofluorescence of the biomolecules and the local concentration of the probe [17]. To solve the limitations, it is necessary to develop a fluorescent molecule capable of providing a dual fluorescence signal to NTR activity in two distinct channels. However, dual emissive fluorescent molecules for NTR detection have been rarely invented [18,19,20].

**Scheme 1:**
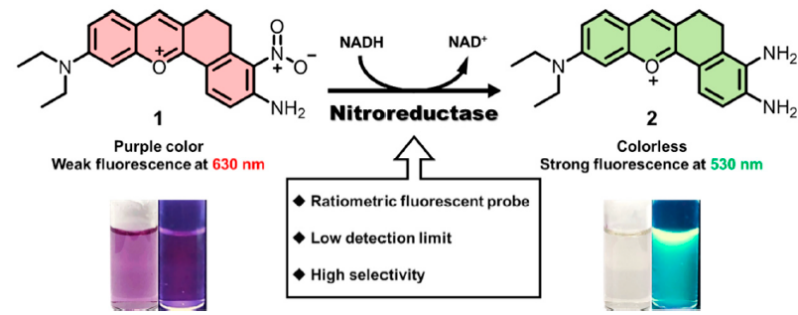
Assay method of probe **1** for NTR activity. Photographs of vials represent the visual and fluorescent colors of probe **1** with and without NTR/NADH in a PBS buffer (pH 7.4)/DMSO (99:1, *v/v*).

In this work, we presented a ratiometric fluorescent molecule (probe **1**) for the assay of NTR activity in the living system. As illustrated in Scheme 1, the nitro group of probe **1** is rapidly reduced to an amino group towards NTR activity, which generates a dual emission change between 630 nm and 530 nm with a solution color change from purple to colorless. Moreover, the dual emission change in living cells can be monitored at two distinct red (615–800 nm) and green (493–550 nm) channels using an excitation laser of 488 nm from a confocal fluorescence microscope. Thus, we suggested that the fluorescent probe **1** can allow a detection of NTR activity in living cells by the ratiometric method.

## 2. Results and Discussion

### 2.1. Synthetic Route of Probe ***1***

**Scheme 2:**
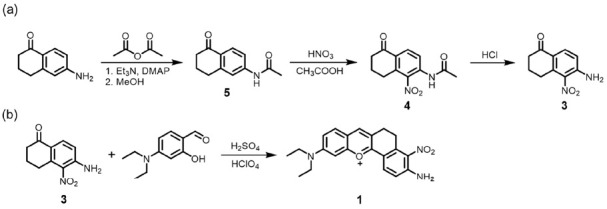
Synthetic routes of (**a**) precursors **3**, **4**, **5** and (**b**) probe **1**.

Probe **1** was devised based on a locked-flavylium fluorophore skeleton with a nitro group sensitive to NTR activity. The flavylium fluorophore was chosen because it can display a ratiometric fluorescence response based on an internal charge transfer (ICT) process. In addition, the locked-flavylium can prevent non-radioactive decay, thereby improving fluorescence emission efficiency [21]. The precursors **3**, **4** and **5** were synthesized by adopting the procedure reported in the previous study (Scheme 2a) [22]. Probe **1** was newly synthesized by a condensation reaction of precursor **3** and 4-(diethylamino)salicylaldehyde in a strong acid condition using H_2_SO_4_ and HClO_4_ with a high yield of 90% (Scheme 2b). The chemical structure of probe **1** was clearly identified by the ^1^H- and ^13^C-NMR spectroscopy, and HR-ESI mass spectrometry (Figures S5–S7).

### 2.2. Sensing Mechanism of Probe ***1***

**Scheme 3:**
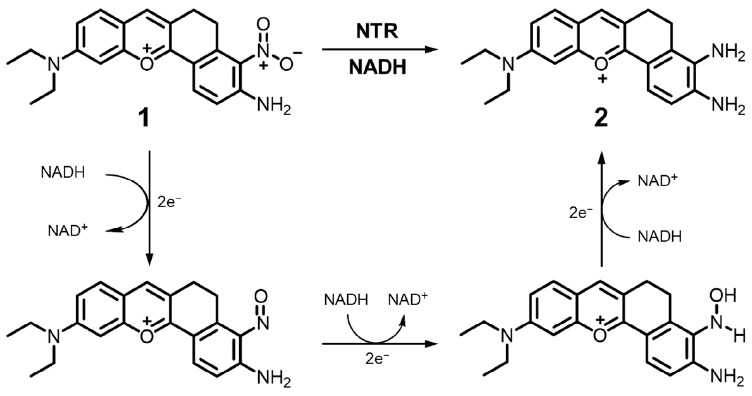
The reduction mechanism of probe **1** by NTR and NADH.

The assay mechanism of probe **1** for an NTR-mediated reaction was shown in Scheme 3. The nitroaromatic moiety of probe **1** is recognized by the NTR enzyme and reduced to the corresponding amine compound **2** using NADH as the electron source [23]. Probe **1** receives two electrons from NADH, electron donor, and converts into nitrosoaromatic intermediate. Sequentially, this intermediate receives more electrons from NADH and reduced to the amine compound **2**, through the hydroxylamine intermediate.

### 2.3. Photophysical Properties of Probe ***1***

**Figure 1:**
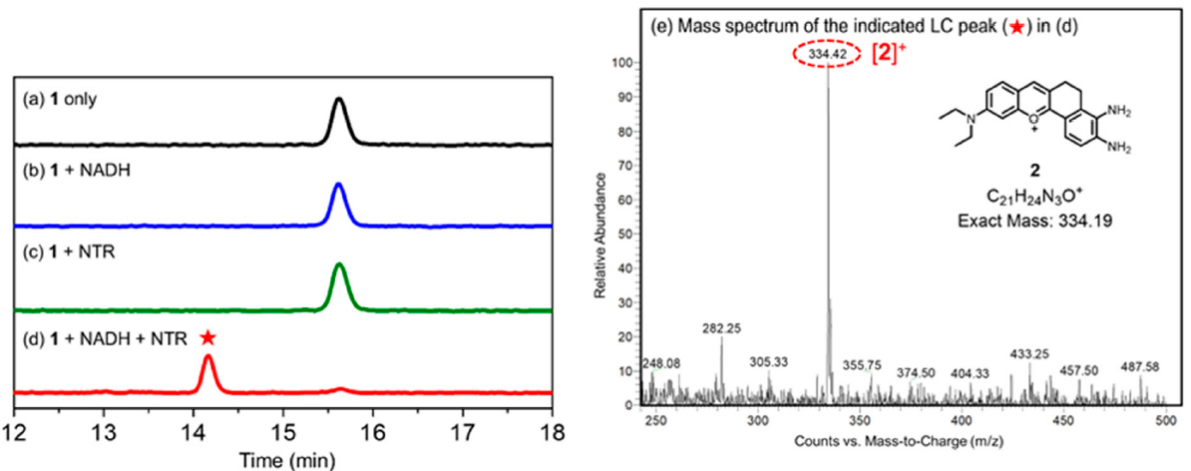
HPLC analyses of (**a**) probe **1**, (**b**) **1**+NADH, (**c**) **1**+NTR, and (**d**) **1**+NADH+NTR. HPLC peaks were detected by recording absorbance at 450 nm. (**e**) ESI-MS spectrum of the eluent at 14.1 min (★).

A production of compound **2** from probe **1** upon exposure to the NTR and NADH was clearly detected by HPLC and ESI-MS spectrometry. In Figure 1, probe **1** shows a LC peak at 15.6 min and is unaffected when NTR or NADH is present independently (Figure 1a–c). However, after incubating probe **1** with NTR and NADH, the LC peak was almost reduced and a new LC peak was detected at 14.1 min (Figure 1d). In addition, the eluent at 14.1 min was analyzed by ESI-MS, resulting in a mass peak at 334.42 m/z congruent to compound **2**. From this analysis, it was confirmed that the operation principle of probe **1** for detection of NTR activity is due to the formation of compound **2** by NTR activity.

**Figure 2:**
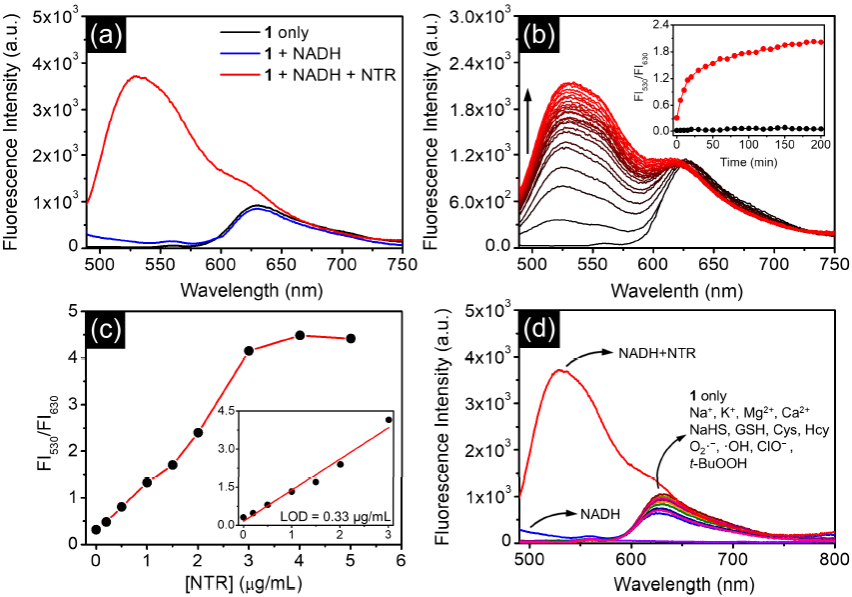
(**a**) Fluorescence spectra of probe **1** (10 μM) with and without NTR (2.0 μg/mL) and NADH (300 μM). (**b**) Time-dependent fluorescence change. Inset: fluorescence intensity ratio vs. time; the black and red lines indicated fluorescence ratio of the probe without and with NTR activity. (**c**) Fluorescence intensity ratio vs. NTR concentrations containing NADH (300 μM). Inset showed a linear correlation with the NTR concentration in the range of 0–3 μg/mL. (**d**) Fluorescence responses to NADH (300 μM), metal ions (1 mM, respectively), thiols (1 mM, respectively), ROS (100 μM, respectively), and NADH (300 μM) + NTR (2 μg/mL). All data were obtained in a PBS buffer (10 mM, pH 7.4)/DMSO (99:1, *v/v*) with 90 min incubation at 37 °C. λ_ex_ = 470 nm.

The absorption and fluorescent emission responses of probe **1** to NTR activity were observed in a PBS buffer (10 mM, pH 7.4)/DMSO (99:1, *v*/*v*) with 90 min incubation at 37 °C. Probe **1** showed the broad absorption and fluorescent emission bands around 570 and 630 nm, respectively (Figure S1 and Figure 2a). However, when probe **1** was incubated with NTR and NADH, the absorption around 570 nm was diminished and new absorption around 450 nm was increased (Figure S1). In addition, upon excitation at 470 nm, new fluorescence emission at 530 nm and shoulder emission at 630 nm were detected (Figure 2a). The absorption and emission changes were also investigated in a course of time after addition of NTR and NADH (Figure 2b and Figure S2). The probe displayed a remarkable fluorescence increase at 530 nm with a constant fluorescence intensity at 630 nm, giving a ratiometric manner between the fluorescence intensities at 530 and 630 nm (Figure 2b).

Such a ratiometric change was saturated in approximately 90 min. Additionally, the ratiometric fluorescence was linearly increased in the NTR concentration range of 0–3 μg/mL, indicting a limit of detection as 0.33 μg/mL (Figure 2c) [24]. For the assessment of the specificity of probe **1** for NTR-mediated enzyme reactions, the absorption and emission was investigated towards biologically interfering biospecies including metals, thiols, reactive oxygen species (ROS), and NADH (Figure 2d and Figure S3). Only the combination of NTR and NADH caused selective absorption and fluorescence changes in contrast to other test analytes.

**Figure 3:**
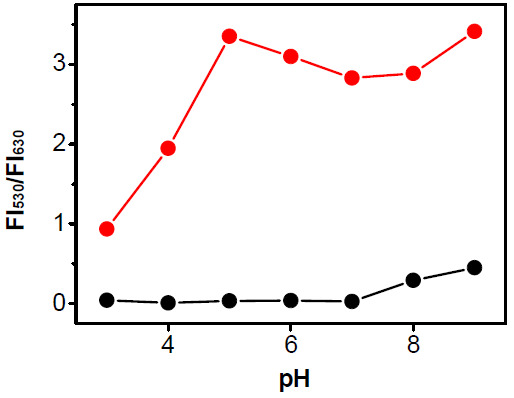
The pH effect on the fluorescence response of probe **1** (10 μM) with (red) and without (black) NTR (2 μg/mL) and NADH (300 μM). All data were obtained in pH buffer containing 10% of DMSO with 90 min incubation at 37 °C. λ_ex_ = 470 nm.

In addition, to investigate the ability of probe **1** to detect NTR activity as a function of pH, a change in fluorescence intensity ratio was observed in various pH solutions (Figure 3). For NTR activity, probe **1** significantly increased the fluorescence intensity ratio, especially at pH 6–9. Meanwhile, without the NTR activity, probe **1** showed a very low fluorescence intensity ratio. Thus, it became more solid that probe **1** can be used to analyze NTR activity in a living system.

In order to further confirm that the fluorescence change of probe **1** results from NTR activity, we performed an inhibition assay using dicoumarol, known as a competitive inhibitor of NADH-dependent enzymes [25,26,27]. At different concentrations of dicoumarol (0–400 μM), probe **1** presented a dose-dependent decrease in the fluorescence intensity ratio (Figure S4). This result firmly demonstrated that the fluorescence ratio changes from probe **1** was caused by NTR activity.

### 2.4. 3-(4,5-Dimethylthiazol-2-yl)-2,5-Diphenyltetrazolium Bromide (MTT) Assay

**Figure 4:**
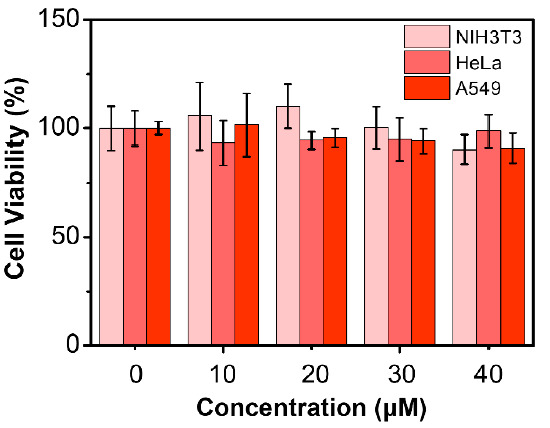
Cytotoxicity of probe **1** to NIH3T3, HeLa and A549 cells. Cells were incubated with various concentrations of probe **1** for 6 h, respectively. Error bars displayed mean ± standard deviation (s.d., *n* = 3).

Prior to confocal microscopic analyses, the cell viability in the presence of probe **1** was confirmed by conducting an MTT assay. In Figure 4, the probe revealed no toxicity in non-cancerous fibroblast NIH3T3, cervical cancer HeLa and lung cancer A549 cells. It implied that probe **1** is highly biocompatible in living cells.

### 2.5. Biological Applications of Probe ***1*** Using Confocal Microscopy

**Figure 5:**
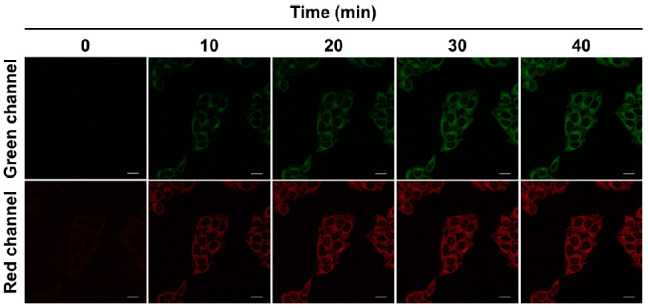
Cellular uptake of probe **1** over time. HeLa cells were incubated with probe **1** (15 μM) for 0–40 min. Images were obtained with confocal microscopy adopting an excitation laser at 488 nm with band-pass filters for green (493–550 nm) and red (615–800 nm) channels. Scale bar: 20 μm.

To investigate the cellular uptake ability of probe **1**, confocal microscopy experiments of probe **1** in HeLa cells were performed at different incubation time sets (0–40 min). The dual image was recorded by adopting an excitation laser at 488 nm with band-pass filters for green (493–550 nm) and red (615–800 nm) channels. In Figure 5, the fluorescence intensity of the red channel turned on an early and then a green channel gradually increased. Moreover, both fluorescence intensities gradually increased with the incubation time and became stable at 30 min incubation. Here, the red image is derived from the probe **1′**s fluorescence penetrated into the cells. However, the green image presented the fluorophore generated from probe **1** supposedly mediated by cellular NTR activity. Based on these results, we propose that probe **1** readily entered living cells and generated a new fluorophore, probably compound **2**, by cellular NTR activity and is optimal at 30 min of incubation.

**Figure 6:**
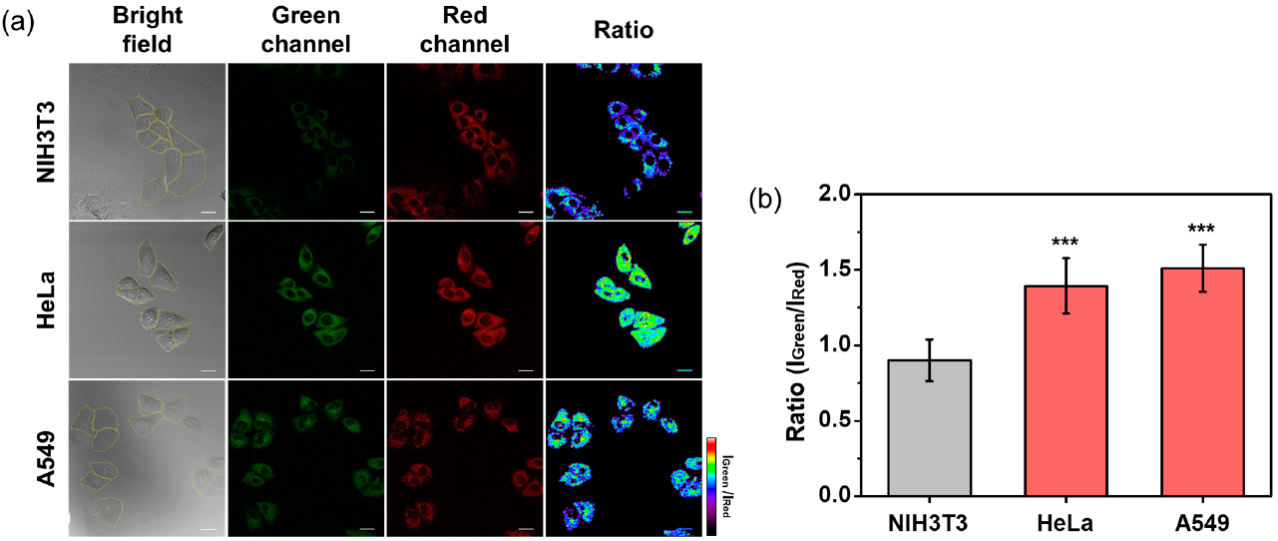
(**a**) Confocal microscopy images for non-cancerous cells (NIH3T3) and cancer cells (HeLa and A549) treated with probe **1** (15μM) for 30min, respectively. Images were obtained by adopting an excitation laser at 488 nm with band-pass filters for green (493–550 nm) and red (615–800 nm) channels. Scale bar: 20 μm. (**b**) Quantification of the fluorescence intensity ratio in the cells. Fluorescence intensities were quantified from the cells (yellow circles, cell counts = 10) in the image by an ImageJ software. Error bars displayed mean ± standard deviation. Asterisks indicate statistically significant changes (*** *p* value < 0.001).

Leveraging the ability of probe **1** for NTR-mediated reaction in a dual manner, NTR activity was compared in non-cancerous fibroblasts NIH3T3, cervical cancer HeLa and lung cancer A549 cells (Figure 6). Fluorescence intensity ratio (I_Green_/I_Red_) in the cell image could be obtained from the red and green channels. The red fluorescence intensity was similar in all the tested cell lines, but the green fluorescence intensity supposedly sensitive to NTR activity was significantly higher in HeLa and A549 cancer cells than in NIH3T3 non-cancerous cells. As a result, the pseudo-colored ratio images clearly suggested a distinctly increased NTR activity in cancer cells compared to non-cancerous cells. It was also a similar tendency for NTR activity in a previously reported study [20]. Based on these results, it was assumed that NTR activity in cells would be a potential biomarker for early diagnose of cancer cells. Here, probe **1** will be a useful assay method for NTR activity in living systems based on self-calibrated ratiometric fluorescence measurements.

## 3. Materials and Methods

### 3.1. Materials and Instrumentation

All chemical reagents used for the synthesis were obtained from Sigma-Aldrich (St. Louis, MO, USA), Alfa (Alfa, Heysham, LA3 2XY, Lancashire, United Kingdom), TCI (Tokyo, Japan). Optical spectra, including absorption and fluorescence, were obtained using on UV-2600 (Shimadzu Corporation, Kyoto, Japan) and RF-6000 (Shimadzu Corporation, Kyoto, Japan) spectrophotometers. Excitation wavelength was 470 nm and the excitation and emission slit widths were 10 nm, respectively. Nuclear magnetic resonance spectra were obtained at Bruker 500 MHz NMR. High-resolution mass spectroscopic analyses (HR-ESI-Mass) were recorded on a liquid chromatography mass spectrometer (LC/MS) at the Korea Basic Science Institute (Seoul, Korea). HPLC analyses were performed by HPLC (Shimadzu LC 6AD, Kyoto, Japan.) equipped with a Thermo Scientific Acclain™ 120 C18 (3μm, 120 Å, 2.1 × 150mm) column (flow rate: 0.5mL/min; mobile phase: buffer A-water with 0.1% *v/v* TFA and buffer B-acetonitrile with 0.1% *v/v* TFA). For pH effect test, 0.1 M citric acid-0.2 M Na_2_HPO_4_ for pH 2–8 and 0.1 M Na_2_CO_3_-0.1 M Na_2_HCO_3_ for pH 9–11 were used [28].

### 3.2. Cell Culture

Human cervical cancer cells (HeLa) were cultured in Dulbecco’s modified Eagle’s medium (DMEM) containing 10% Gibco^®^ fetal bovine serum (FBS) and 100 U/mL penicillin-streptomycin. Adenocarcinoma human alveolar basal epithelial cells (A549) were cultured in Roswell Park Memorial Institute (RPMI) 1640 medium containing 10% FBS and 100 U/mL penicillin-streptomycin. Mouse embryonic fibroblasts (NIH3T3) were cultured in DMEM medium containing 10% Gibco^®^ bovine calf serum (BCS) and 100 U/mL penicillin-streptomycin. Cells were seeded at 10^5^ per dish 2 days prior to microscopic experiments and transferred to cover glass bottom dishes. Cells were incubated at 37 °C with a 5% (*v/v*) CO_2_ contained air. Cell lines were obtained from Korea Cell Line Bank (Seoul, Korea). DMEM, RPMI, FBS, trypsin 0.25%-EDTA and penicillin-streptomycin used in cell experiments were obtained from BIOWEST (Cholet, France). The clear and adhesion-typed confocal dishes (diameter = 35 mm) were used from SPL (Phocheon-si, gyeonggi-do, Korea).

### 3.3. Synthesis of Probe ***1***

Precursor **3** was prepared according to literature procedure [22]. The **3** (0.45 g, 2.2 mmol) and 4-(diethylamino) salicylaldehyde (0.43 g, 2.2 mmol) were dissolved in sulfuric acid (40 mL). The solution was stirred and refluxed for 5 h under a nitrogen gas, cooled to RT and poured into ice, and 20 mL perchloric acid was slowly added. The product was extracted three times with dichloromethane (DCM), and anhydrous sodium sulfate was used to remove residual water in the collected DCM solution. The solvent was evaporated and a recrystallization using methanol/hexane afforded **1** as a dark green powder (0.73 g, 90%). HR-ESI-MS m/z [M + H]^+^ calc. 364.17, obs. 364.1658. ^1^H-NMR (500 MHz, DMSO-*d*_6_): δ (ppm) 1.23 (t, *J* = 6.9 Hz, 6H), 2.99 (s, 4H), 3.72–3.60 (m, 4H), 7.05 (d, *J* = 9.0 Hz, 1H), 7.23 (s, 1H), 7.37 (d, *J* = 7.7 Hz, 1H), 7.60 (s, 2H), 7.88 (d, *J* = 9.3 Hz, 1H), 8.17 (d, *J* = 9.0 Hz, 1H), 8.56 (s, 1H). ^13^C-NMR (125 MHz, DMSO-*d*_6_): δ (ppm) 163.3, 158.0, 155.0, 148.0, 147.3, 139.9, 134.9, 131.9, 130.4, 119.6, 117.4, 117.3, 117.2, 114.2, 96.2, 45.7, 24.4, 23.6, 12.9.

## 4. Conclusions

We developed an NTR-sensitive ratiometric fluorescence probe **1**, composed of locked-flavylium dye and nitroaromatic moiety, and demonstrated that probe **1** is highly biocompatible and sensitive to NTR activity in vitro and supposedly also in living cells. Moreover, probe **1** gave rise to a significantly increased fluorescence intensity ratio in A549 and HeLa cancer cells than in NIH3T3 non-cancerous cells. We propose that cellular NTR activity could be a potential target in the fields of early diagnosis, drug discovery, and cancer therapy, moreover it is potentially well detectable by our newly developed probe.

## Data Availability

The data presented in this study are available in this article.

## Supplementary Materials

The following are available online, Figure S1. UV/Vis absorption spectra of probe **1** with or without NTR and NADH. Figure S2. Time-dependent absorption changes of probe **1** (10 μM) in the presence of NTR (2.0 μg/mL) and NADH (300 μM). Figure S3. UV/Vis absorption spectra of probe **1** (10 μM) toward NADH (300 μM), metal ions (Na^+^, Mg^2+^, K^+^, and Ca^2+^; 1 mM, respectively), thiols (NaHS, GSH, Cys, and Hcy; 1 mM, respectively), ROS (O_2_^−^, ·OH, t-BuOOH, and ClO^−^; 100 μM, respectively), and NADH (300 μM)+NTR (2 μg/mL). Figure S4. Inhibition assay of NTR activity using various concentrations of dicoumarol based on fluorescence ratio (FI_530_/FI_630_) of probe **1** (10 μM). The fluorescence intensity ratio from probe **1** were measured to NTR activity (2 μg/mL of NTR and 300 μM of NADH) at different concentrations of dicoumarol (0, 100, 200, and 400 μM). Figure S5. ^1^H-NMR spectrum of **1** in DMSO-*d*_6_. Figure S6. ^13^C-NMR spectrum of **1** in DMSO-*d*_6_. Figure S7. HR-ESI-MS spectrum of **1**.

## Sample Availability

Not available.
