# Supplementary material for: Ratiometric Fluorescence Assay for Nitroreductase Activity: Locked-Flavylium Fluorophore as a NTR-Sensitive Molecular Probe
Source: Molecules. 2021 Feb 19;26(4):1088. doi: 10.3390/molecules26041088 (PMC7923055; doi:10.3390/molecules26041088)
Supplement: Supplementary file 1 [file molecules-26-01088-s001.pdf]

Supplementary Information

Ratiometric Fluorescence Assay for Nitroreductase Activity: Locked-Flavylium Fluorophore as a NTR-sensitive Molecular Probe

Su Jung Kim[1‡], Jung Won Yoon[1‡], Shin A Yoon[1‡], Min Hee Lee[1*]

[1]Department of Chemistry, Sookmyung Women's University, Seoul 04310, Korea
* Correspondence: minheelee@sookmyung.ac.kr; Tel: +82-2-2077-7815
[‡]These authors are equally contributed to this work

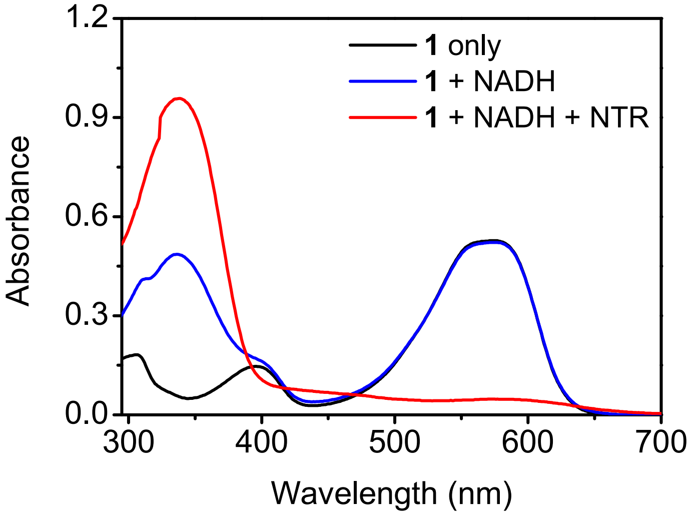

Figure S1. UV/Vis absorption spectra of probe **1** with or without NTR and NADH. All data were obtained in PBS buffer (10 mM, pH 7.4)/DMSO (99:1, v/v) with an incubation of 90 min at 37 °C. λ_{ex} = 470 nm.

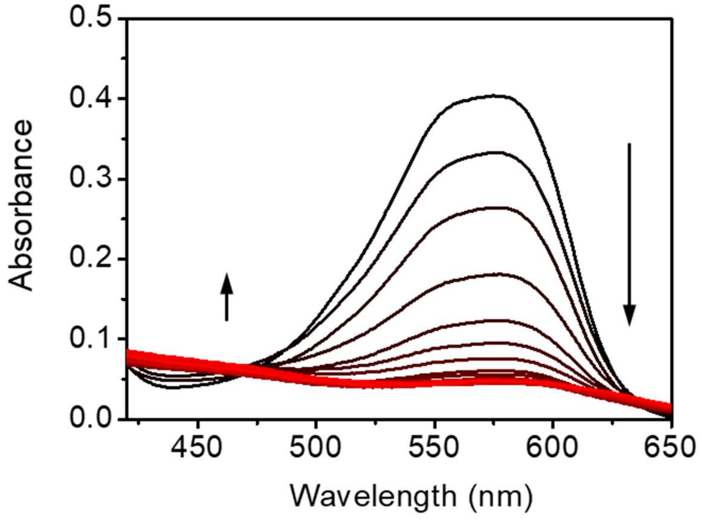

Figure S2. Time-dependent absorption changes of probe **1** (10 μM) in the presence of NTR (2.0 μg/mL) and NADH (300 μM). All data were obtained in PBS buffer (10 mM, pH 7.4)/DMSO (99:1, v/v) with an incubation of 90 min at 37 °C. λ_{ex} = 470 nm.

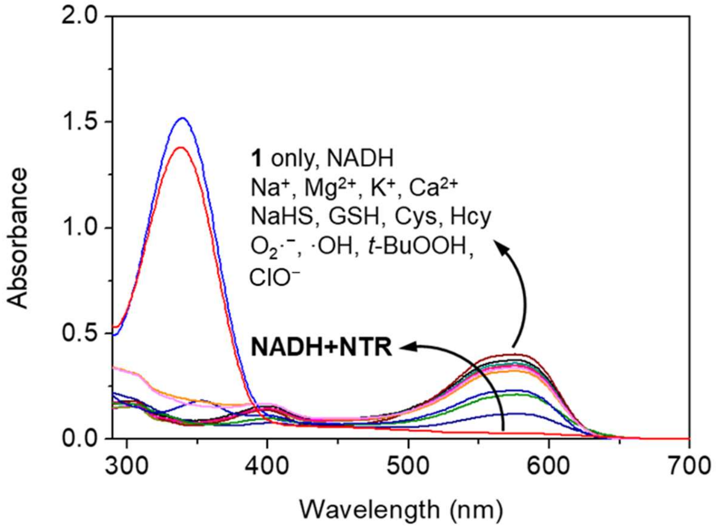

Figure S3. UV/Vis absorption spectra of probe **1** (10 μM) toward NADH (300 μM), metal ions (Na^+, Mg^{2+}, K^+, and Ca^{2+}; 1 mM, respectively), thiols (NaHS, GSH, Cys, and Hcy; 1 mM, respectively), ROS ($O_2{\cdot}^-$, ·OH, *t*-BuOOH, and ClO^-; 100 μM, respectively), and NADH (300 μM)+NTR (2 μg/mL). All data were obtained in PBS buffer (10 mM, pH 7.4)/DMSO (99:1, v/v) with an incubation of 90 min at 37 °C. λ_{ex} = 470 nm.

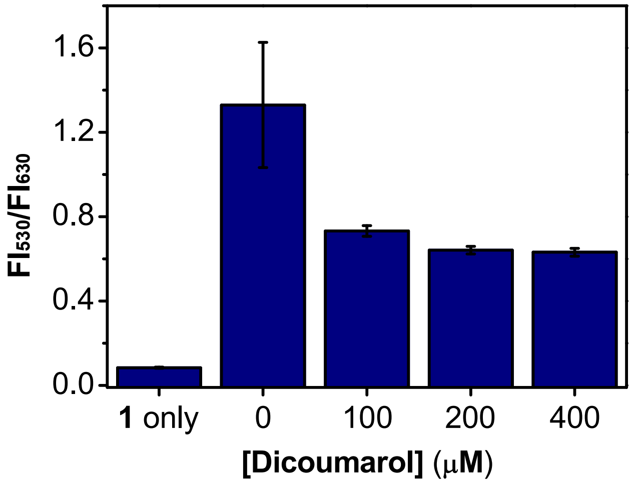

Figure S4. Inhibition assay of NTR activity using various concentrations of dicoumarol based on fluorescence ratio (FI_{530}/FI_{630}) of probe **1** (10 μM). The fluorescence intensity ratio from probe **1** was measured to NTR activity (2 μg/mL of NTR and 300 μM of NADH) at different concentrations of dicoumarol (0, 100, 200 and 400 μM). All data were obtained in PBS buffer (10 mM, pH 7.4)/DMSO (9:1, v/v) with 90 min incubation at 37 °C. λ_{ex} = 470 nm. Error bars displayed mean ± standard deviation (s.d., n = 3).

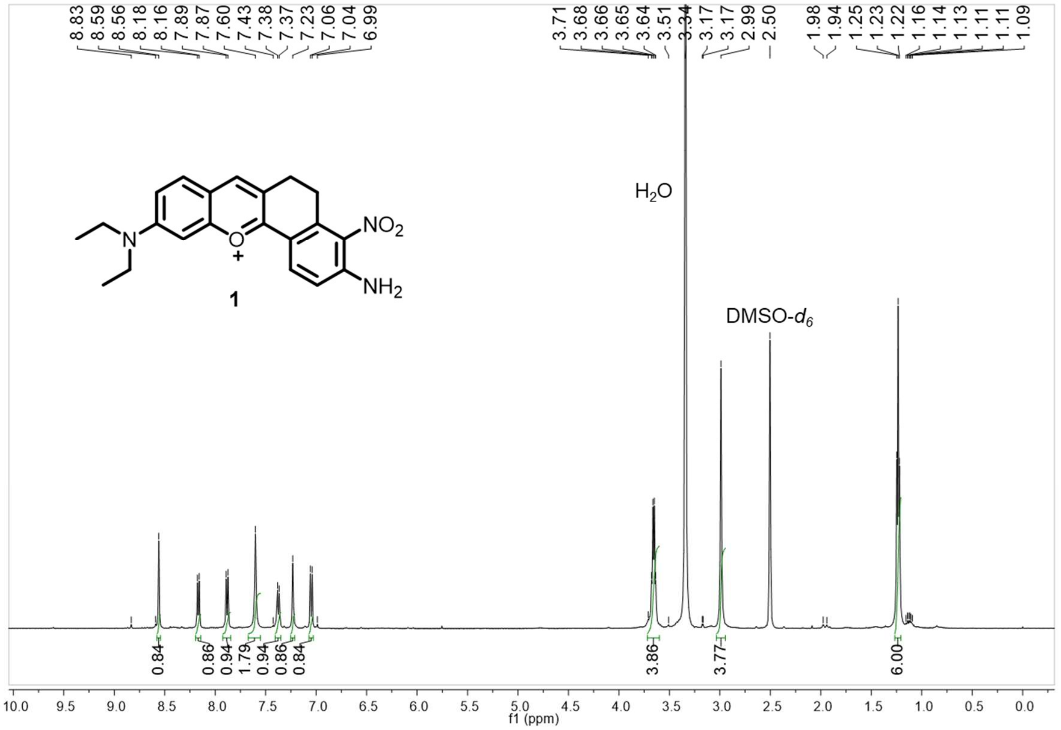

Figure S5. ^{1}H NMR spectrum of **1** in DMSO-d_6.

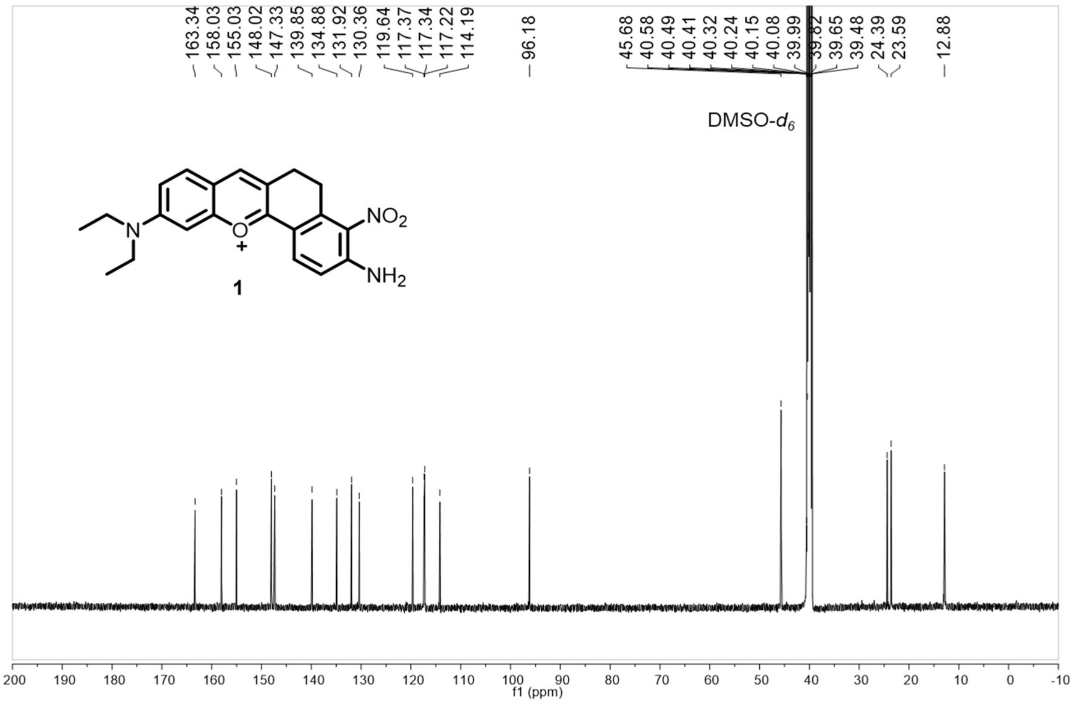

Figure S6. ^{13}C NMR spectrum of **1** in DMSO-d_6.

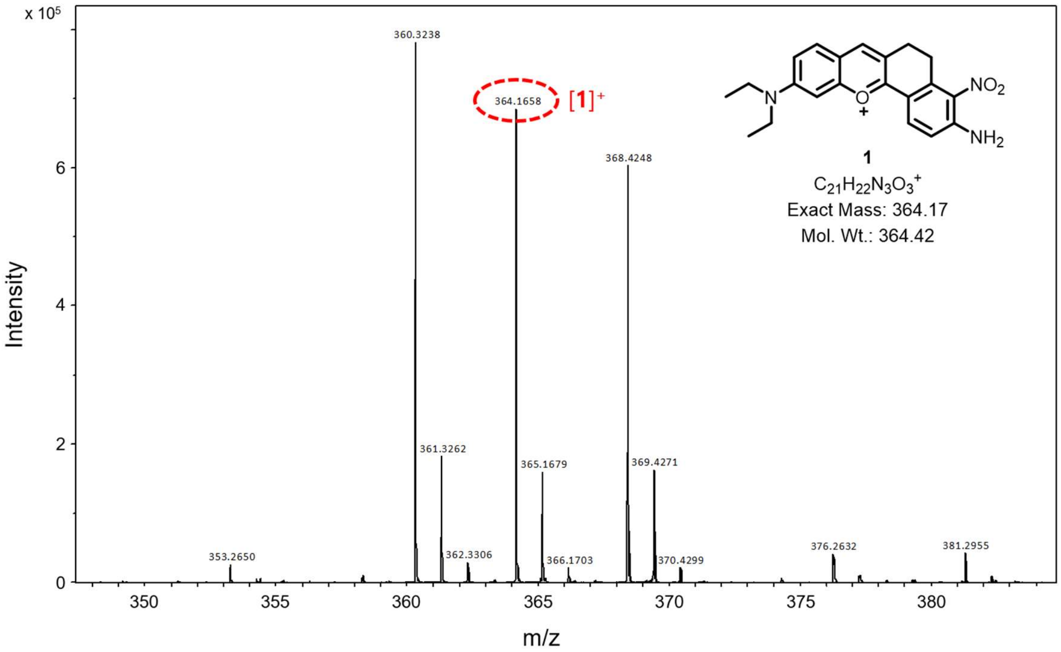

Figure S7. HR-ESI-MS spectrum of **1**.
